# Current Thinking on *Eimeria* spp. in Poultry and Potential for Advancements in Control

**DOI:** 10.3390/ani15030424

**Published:** 2025-02-03

**Authors:** Kenneth Bafundo

**Affiliations:** Poultry Diagnostic and Research Center, College of Veterinary Medicine, University of Georgia, Athens, GA 30602, USA; ken.bafundo@gmail.com

## 1. Introduction

The significance of *Eimeria* infections in chicken production cannot be understated. The infections are strikingly ubiquitous, with the strong likelihood that every commercial flock of chickens encounters one or more infections caused by these parasites. Some flocks, no doubt, experience only mild effects such as slower growth and compromised efficiency, while others undergo severe disease symptoms characterized by performance losses, hemorrhage, and elevated mortality. Parasite development within the intestine often leads to complications brought about by the presence of other pathogens, and in turn, complicated disease symptoms and animal welfare issues are often initiated by coccidial infections [1]. From a financial perspective, estimates show that annual global losses in the range of USD 14 billion routinely occur [2]. Moreover, global events such as the COVID-19 pandemic and the war in Ukraine have likely exacerbated these losses by more than 20% [3]. In order to meet the world’s growing demand for high-quality protein, the years ahead will likely see expansive growth in both broiler and egg production. Commensurate with this growth will be the ever-present economic burden of *Eimeria* infections.

Since the 1940s, commercial chicken producers have relied on chemotherapy as the primary means of managing coccidial infections. Early on, chemically synthesized coccidiostats were commonly used to limit mortality, as broiler producers of the 1960s focused on the control of the hemorrhagic coccidia *E. tenella* and *E. necatrix*. During the 1970s and 1980s, several polyether ionophores were introduced, providing broad, effective control of *Eimeria* spp., allowing the industry to benefit from better intestinal health and, along with it, improved growth and feed efficiency. However, beginning in the mid-1990s, research on new anticoccidials waned, and since that point in time, only one anticoccidial product has been developed for coccidiosis in broiler chickens [4].

In the past two decades, the availability and use of anticoccidial products has also been affected by regional public demand for animals reared in the absence of chemotherapeutic agents [1,5]. In the EU, for example, several anticoccidial products have been banned, and the No Antibiotics Ever (NAE) movement in the United States prohibits the use of ionophore anticoccidials in broilers reared for that market segment [1,6]. Together, lack of research and consumer pressure on existing anticoccidial agents has forced the use of older, less resilient anticoccidials in today’s production systems, thereby requiring additional support in order for optimal growth and efficiency to be achieved [6].

In order to break the cycle of continuous drug usage, live coccidiosis vaccines are often applied. Successful vaccine usage relies upon specialized management considerations (vaccine storage and application, litter and house management, etc.) that foster oocyst survival and allow for the re-exposure of birds to these infective forms. When these details are effectively addressed, vaccination has the potential to become a reasonable alternative to chemotherapy. Because vaccines contain drug-sensitive coccidia, modification of the drug sensitivity profiles of coccidia from vaccinated houses is known to occur. This approach is often used to improve the performance of many anticoccidial drugs once the return to chemotherapy occurs.

Of course, live vaccines have well-recognized effects on intestinal integrity, and producers often express concerns about negative performance results during vaccine usage [7]. The “bio-shuttle program”, utilizing both vaccine application and short-term anticoccidial use (during approximately 10 days of peak coccidiosis challenge), limits the negative effects of vaccinal *Eimeria* replication during critical growth periods [8], thereby allowing vaccination to be more cost-competitive with standard chemotherapeutic approaches.

Although interest in a non-living vaccine remains very high, progress toward this objective has been difficult. Several laboratories have carried out pen and field trials with a variety of *Eimeria* antigens or genes for various antigens. A number of different application techniques has also been evaluated [4]. To date, however, none of these approaches has exceeded live vaccination in terms of efficacy, bird performance, ease of application, and/or cost effectiveness. It therefore appears that vaccination with live parasites will remain the most practical means of immunological protection for the foreseeable future.

Given this scenario, it should be clear that many poultry veterinarians express concern about the current and future status of coccidial infection [9]. These specialists recognize the need for technological advances for improved *Eimeria* control, a fact which underscores the need for a better understanding of these parasites and the infections that they produce. The enclosed Special Issue is devoted to current research on coccidiosis in chickens, with the hope that it will address some of the needs and information gaps described above.

The papers presented herein deal with several different aspects of *Eimeria* infections. The topics range from studies on the nutritional effects of *Eimeria* infections in broilers and layers to new methods of *Eimeria* control that diminish intestinal inflammation. Novel methods of oocyst speciation using artificial intelligence are also presented, along with reports of the new *Eimeria* species identified in Romania. The effects of coccidial infection on the intestinal microflora, a new and significant area of research, are also addressed in three separate contributions. Additionally, studies on the identification and testing of newly characterized antigens for inclusion in future vaccines are also included.

## 2. An Overview of Published Articles

Traditionally, the broiler chicken has been the standard focus of investigations dealing with *Eimeria* infections and host nutrition. In fact, nutritional studies in broilers have become one of the more commonly addressed topics in recent years. Varying greatly from this usual approach, the review of Sharma and Kim (Contribution 1) evaluated the effects of coccidial infections in laying fowl, and, in particular, outlined the significance of *Eimeria* infections in the developing pullet, in the laying hen, as well as the effects on egg production. In addition to describing the disease in the pullet/layer and noting the standard methods of control, the comprehensive nature of this work references basic nutritional research gleaned from studies in several avian species, but also presents new results found in production hens. The work clearly emphasizes the *Eimeria*-related effects that influence hen physiology and egg production and offers recommendations for nutritional strategies that could potentially limit these effects. This is an exceptionally cogent review that provides insights into layer health and egg production that have been lacking in the published literature.

Given the growing significance of live oocyst vaccination in broiler production, the work of Myers and Rochell (Contribution 2) examined the effects of fully pathogenic *Eimeria* vaccination on energy utilization when compared to birds receiving anticoccidial medication. When compared to medicated treatments in a 43-day trial, vaccination impaired nutrient and energy digestibility, body weight, and carcass yield when measured at days 18 and 31. In addition, the effects of vaccination on feed conversion were dependent upon energy level in starter feeds, and these effects were evident both at 18 days and in all subsequent feeding periods. The greatest negative effects were recorded for treatments receiving the highest energy levels in starter feed. Consequently, the authors explain that compensating for impaired energy utilization during vaccination by increasing dietary energy content in starter feed is ineffective. Clearly, additional research is required in order to understand the mechanisms of *Eimeria*-induced lipid malabsorption that extend beyond the limitations of energy availability.

The global emphasis on the reduction in antibiotic usage in poultry has led to the investigation and application of several new classes of compounds that influence the development of *Eimeria* and the adverse effects that these infections produce. The studies of Fellici et al. (Contribution 3) applied novel in vitro techniques to determine whether certain essential oils (thyme, oregano, and garlic) affect host cell penetration by *E. tenella* sporozoites and the subsequent development of *E. tenella* schizonts. Their work demonstrated that these oils decrease sporozoite invasion in vitro, thereby limiting the development of *E. tenella* schizonts. In some cases, both the size and number of schizonts, factors that are strongly associated with the pathogenicity of *E. tenella*, were significantly reduced. It was suggested that these in vitro techniques could be applied to the identification and development of new anticoccidial entities.

Sadoris et al. (Contribution 4) evaluated combinations of yucca (*Yucca schidigera*) and quillaja (*Quillaja saponaria*) in *Eimeria*-challenged broilers to determine their effects on intestinal permeability and immune responsiveness. While oocyst production and lesion scores were significantly reduced by oral administration of this combination, the saponin formula also maintained the integrity of the intestinal epithelium. *Eimeria*-induced cytokines (IFN-γ, IL-17, and IL-10) were reduced in the infected treatment group receiving saponins when compared to infected controls, and these levels did not differ from those recorded for the non-infected control group. This observation helps to explain the reported effects of saponins on the limitations of inflammation during and following coccidial infections.

Villar-Patino et al. (Contribution 5) studied the effects of extracts of Alliaceae (*Allium*) during coccidial infection by demonstrating improvements in feed conversion and changes in the microbial composition of the gut during feeding.

To better understand the effects of *Eimeria* infections on the intestinal microflora, Wang et al. (Contribution 6) repeatedly infected broiler chicks with low doses of *E. mitis,* producing birds that were immunologically protected from *E. mitis* challenge. Following this procedure, birds were monitored for changes in intestinal health and the composition of the intestinal microflora. The results indicated that multiple exposures to *E. mitis* increased the numbers of opportunistic bacterial pathogens in the intestine; a generalized decrease in non-pathogenic bacteria also occurred. Both findings were associated with increased levels of dysbacteriosis. Thus, in addition to previously reported impairments of growth performance, *E. mitis* infections, even when produced by repeated low-dose exposures, cause meaningful changes in the intestinal microbiota. The work illustrates the importance of *E. mitis*, a common coccidial parasite of broilers that is often unrecognized during routine *Eimeria* surveillance.

Miska et al. (Contribution 7) studied the effects of *E. maxima* infections on both luminal and mucosal bacterial populations of the intestine over a 14-day period following parasite exposure. The results demonstrated that *E. maxima* affected both alpha and beta diversity in intestinal bacteria, and that these effects commonly occurred at the height of infection. By day 14 post-infection, differences between the infected and control populations were minimal. While *E. maxima* infection increased the levels of *Lactobacillus*, butyrate-producing bacteria, thought to be beneficial in mucosal repair, were more commonly associated with non-infected broilers. The study also showed that luminal and mucosal bacterial populations differed markedly, thus requiring separate analyses of these two populations so that a more complete picture of the ecology of the intestinal microbiota could be presented.

Studies have shown that proteins isolated from the rhoptries of *E. tenella* display characteristics of protective antigens. The work of Li et al. (Contribution 8) investigated *E. tenella* ROP27 (EtROP27) as a potential component of a subunit coccidiosis vaccine. This rhoptry protein is known to be expressed at all stages of *E. tenella* development. The protein was produced in a procaryotic expression system, and after purification, it was tested in broilers challenged with *E. tenella*. Administration of EtROP27 was found to increase Ig-Y titers in serum; improvements in body weight gain and reductions in oocyst production, lesion scores, and bloody feces were also observed. While in vivo testing of this protein was limited in scope, these findings illustrate the potential of EtROP27 as a component of a future vaccine.

In most diagnostic situations, speciation of the coccidia involved is required. Traditionally, this has been accomplished through oocyst identification and lesion scoring, but there are inherent shortcomings and time commitments associated with these methodologies [10]. Kellogg et al. (Contribution 9) developed an artificial intelligence-based technique for the accurate identification of *Eimeria* oocysts in fecal samples. The method also has the capability of differentiating sporulated from non-sporulated oocysts of the species examined. The technique relies upon automated image analysis of samples that are then compared to examples of oocysts that have been previously identified as representative of the species. The reported results suggest that this image analysis technique is approximately equivalent to standard methods in terms of the accuracy of identification, but can markedly reduce analysis time compared to manual procedures. Further development of the methodology is in progress so that the minor species and those with smaller oocysts can be more accurately identified. Once completed, these additions will enhance reliability and make the technique an important tool for diagnosticians and researchers in the field.

Using PCR techniques, Coroian et al. (Contribution 10) examined the coccidial species parasitizing free-range chickens in Romania. *Eimeria acervulina* and *E. tenella* were the most commonly identified species, with *E. praecox, E. brunetti,* and *E. mitis* also recognized in several flocks. Surprisingly, *E. maxima* was not identified. Of importance was the recognition of OTUy and OTUz (*E. nagambie* and *E. zaria*, newly recognized species from the chicken) in a number of Romanian flocks. While *E. zaria* has previously been identified in Europe, this work is the first to report OTUy (*E. nagambie*) in a European country. In addition, common helminth infections were also reported, with *Ascaridia, Heterakis,* and *Capillaria* frequently recognized.

## 3. Concluding Comments

As noted above, it is hoped that the papers published in this Special Issue address some of the challenges presented by *Eimeria* infections and potentially fill some of gaps in our knowledge of these important parasites. It is also hoped that this volume will stimulate other researchers to focus their attention on the growing significance of coccidial infections in chickens and direct their efforts toward a better understanding of and potential solutions for this malady. In either case, readers are encouraged to examine these 10 contributions, since each has a place in the modern literature devoted to *Eimeria* in chickens.

## References

[1] Hofacre C.L., Smith J.A., Mathis G.F. (2018). An optimist’s view on limiting necrotic enteritis and maintaining broiler gut health and performance in today’s marketing, food safety and regulatory climate. Poult. Sci..

[2] Blake D.P., Knox J., Dehaeck B., Huntington B., Rathinam T., Ravipati V., Ayoade S., Gilbert W., Adebambo A.O., Jatau I.D. (2020). Re-calculating the cost of coccidiosis in chickens. Vet. Res..

[3] Blake D.P. (2024). *Eimeria* of chickens: The changing face of an old foe. Avian Pathol..

[4] Blake D.P., Marugan-Hernandez V., Tomley F.M. (2021). Spotlight on avian pathology: *Eimeria* and the disease coccidiosis. Avian Pathol..

[5] Cervantes H.M. (2015). Antibiotic-free poultry production: Is it sustainable?. J. Appl. Poult. Res..

[6] Bafundo K.W., Duerr I., McNaughton J.L., Gomez L. (2022). The influence of a quillaja and yucca combination on growth performance and lesion scores of broilers administered chemical anticoccidials or a live coccidiosis vaccine. Int. J. Poult. Sci..

[7] Lee J.T., Eckert N.H., Ameiss K.A., Stevens S.M., Anderson P.N., Anderson S.M., Barri A., McElroy A.P., Danforth H.D., Caldwell D.J. (2011). The effect of dietary protein level on performance characteristics of coccidiosis vaccinated and nonvaccinated broilers following mixed-species *Eimeria* challenge. Poult. Sci..

[8] Mathis G., Schaeffer J., Cookson K., Dickson J., LaVorgna M., Waldrip D. (2014). Effect of lasalocid or salinomycin administration on performance and immunity following coccidia vaccination of commercial broilers. J. Appl. Poult. Res..

[9] United States Animal Health Association (2019). Report of the USAHA Committee on Poultry and Other Avian Species. https://www.usaha.org/upload/Committee/2019Reports/Poultry_and_Other_Avian_Species_.pdf.

[10] Long P.L., Joyner L.P. (1984). Problems in the identification of the species of *Eimeria*. J. Protozool..

